# Postpartum Haemorrhage Burden, Management and Challenges in Malaysia: A Scoping Review and Expert Recommendations for Effective Management

**DOI:** 10.7759/cureus.89915

**Published:** 2025-08-12

**Authors:** Mukhri Hamdan, Carol Lim, Boon Nee Tang, Habibah Abdul Hamid, Vallikannu Narayanan, Nur Azurah Abdul Ghani, Mohd Pazudin Ismail, Veena Selvaratnam, Surbhi Wadhawan, Deepak Mukherjee

**Affiliations:** 1 Department of Obstetrics and Gynaecology, Faculty of Medicine, Universiti Malaya, Kuala Lumpur, MYS; 2 Department of Obstetrics and Gynaecology, Hospital Ampang, Ampang Jaya, MYS; 3 Department of Obstetrics and Gynaecology, Subang Jaya Medical Centre, Subang Jaya, MYS; 4 Department of Obstetrics and Gynaecology, University Putra Malaysia (now Hospital Sultan Abdul Aziz Shah), Serdang, MYS; 5 Department of Obstetrics and Gynaecology, University Malaya Medical Centre (UMMC), Kuala Lumpur, MYS; 6 Department of Obstetrics and Gynaecology, Hospital Universiti Kebangsaan Malaysia Persiaran (HUKM), Kuala Lumpur, MYS; 7 Department of Obstetrics and Gynaecology, Universiti Sains Malaysia (USM), Kubang Kerian, MYS; 8 Department of Hematology, Hospital Ampang, Ampang Jaya, MYS; 9 Medical Affairs, Novo Nordisk Pharma (Malaysia) Sdn. Bhd., Kuala Lumpur, MYS; 10 Clinical, Medical, and Regulatory Affairs, Novo Nordisk Pharma (Malaysia) Sdn. Bhd., Kuala Lumpur, MYS

**Keywords:** early diagnosis, management, patient journey, postpartum haemorrhage, treatment

## Abstract

Postpartum haemorrhage (PPH) and severe postpartum haemorrhage (sPPH) represent significant causes of maternal morbidity and mortality in Southeast Asia, particularly in Malaysia, where PPH remains a leading cause of maternal death. This scoping review systematically examined published literature in English-language, from January 2013 to January 2023 using PubMed and Google Scholar databases, ultimately including 16 studies that met the inclusion criteria for observational and clinical studies in pregnant women from Southeast Asia. The findings reveal substantial challenges in PPH management across the region, including inconsistent diagnostic criteria with varying blood loss thresholds, inadequate awareness among healthcare professionals and patients, and limited access to standardised treatment protocols. Cultural factors significantly impact healthcare-seeking behaviour, such as the majority of Malaysian women preferring traditional healers over medical facilities for PPH symptoms. The review identified critical gaps in blood loss estimation methods, with visual estimation being unreliable and leading to underdiagnosis, while patient intrinsic factors such as uterine atony, retained placenta, and placenta accreta spectrum disorders contribute to treatment complexity. Management approaches varied considerably, with mixed responses to first-line treatments including oxytocin, carbetocin, and misoprostol, while invasive procedures like intrauterine balloon tamponade were commonly employed for refractory cases. The study highlights the urgent need for standardised protocols, improved training of healthcare providers, and enhanced access to novel therapies such as recombinant activated factor VII, recently approved for PPH management. Expert recommendations propose revised diagnostic criteria emphasising clinical signs over absolute blood loss measurements. The review concludes that addressing PPH burden in Southeast Asia requires a multifaceted approach, including improved reporting systems, standardised treatment protocols, enhanced healthcare provider training, and adoption of evidence-based therapies adapted to resource-limited settings.

## Introduction and background

Postpartum haemorrhage (PPH, defined as blood loss of ≥500 mL following a vaginal delivery or ≥1000 mL following caesarean delivery) is a life-threatening obstetrical emergency accounting for approximately a quarter of global maternal deaths [1,2]. It is estimated that 14 million women globally experience PPH annually, resulting in about 70,000 deaths [3]. There is sparse information available on PPH incidence in Southeast Asia. In Thailand, PPH incidence rates are reported to be between 2.4% and 4.35% with 50% PPH-associated maternal deaths in some regions [4]. In India, PPH incidence ranges between 2% and 4% following vaginal delivery and 6% post-caesarean, with PPH being reported as one of the principal causes of maternal death [5]. In 2021, the maternal mortality ratio in Malaysia was reported to be 68.2 per 100,000 live births, with PPH being one of the leading causes of death [6,7].

**Figure 1:**
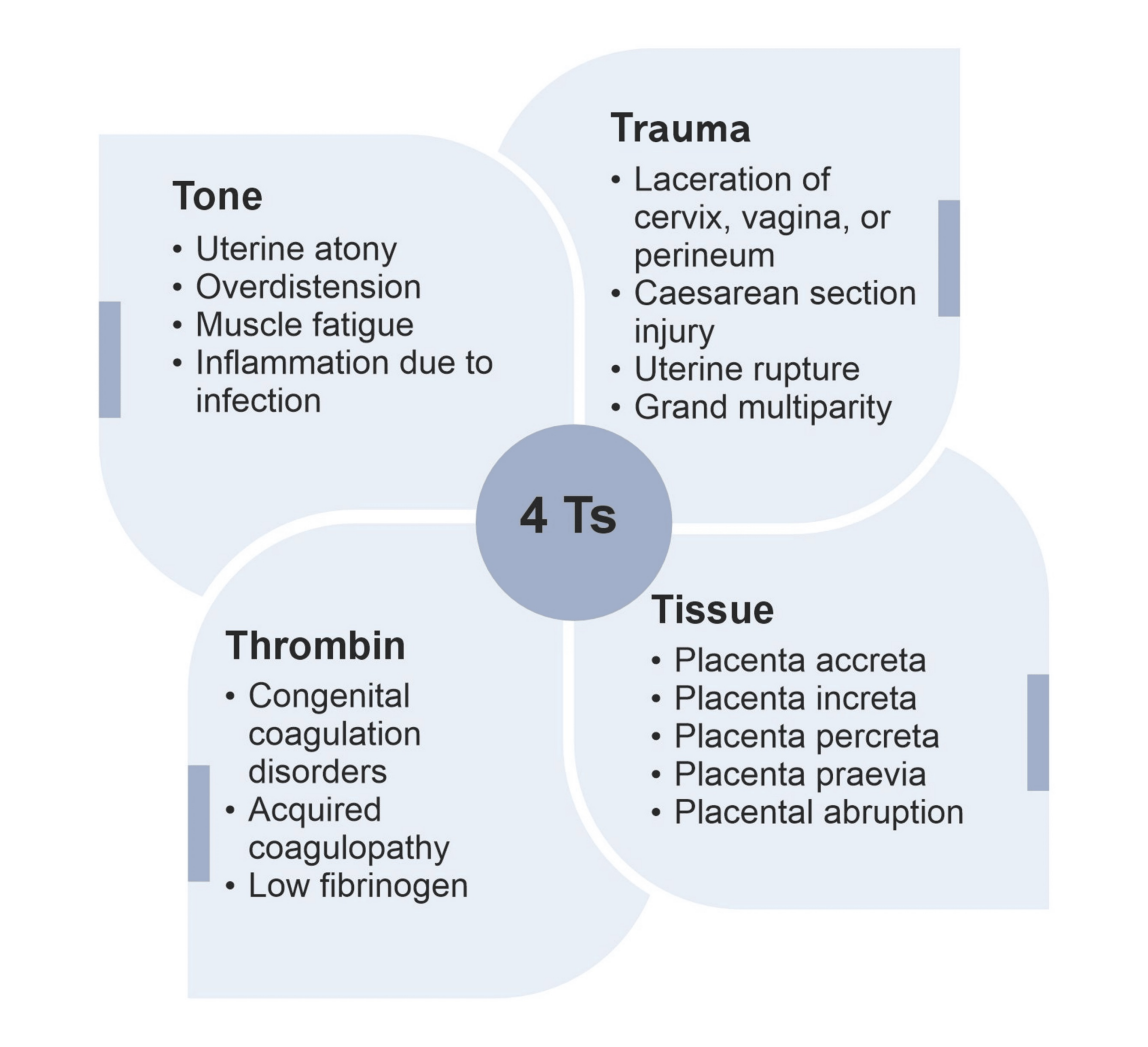
Major Causes of PPH – 4Ts Figure created by the authors based on information from [10] PPH: Postpartum haemorrhage

Poorly managed PPH can rapidly progress to severe PPH (sPPH, defined as blood loss ≥1500 mL or the need for blood transfusion for excessive bleeding at the time of delivery), leading to an increased need for hysterectomy, unpredictable ICU admissions, and prolonged hospital stays [8,9]. An inaccurately estimated blood loss may result in the sPPH not being fully diagnosed. Both PPH and sPPH represent a traumatic childbirth experience, resulting in long-term paternal and maternal psychological impact-postpartum depression, and post-traumatic stress disorder. Assessment of different risk factors for PPH (tone, tissue, trauma, and thrombin levels) (Figure 1) and timely diagnosis is therefore important to ensure prompt management of PPH and prevent maternal mortality and morbidity during childbirth [10]. Maternal morbidities are more common with PPH; in Malaysia, 53.2% of maternal morbidity cases were attributed to PPH [11]. Furthermore, there is limited data supporting the ability of risk-assessment tools commonly used in obstetrics to accurately predict women at risk for PPH [12]. Little is known about the predictors for sPPH, especially after caesarean delivery since the need for peripartum blood transfusion in women undergoing caesarean delivery is reported to be higher than in normal birth [13].

Interventions to treat PPH generally proceed from less to more invasive and include uterotonic medications, compression techniques, invasive procedures including intrauterine balloon tamponade (IUBT), and surgeries. Interventions may also involve adjunctive therapies, such as blood and fluid replacement and/or an anti-shock garment, to treat the blood loss and other sequelae that result from PPH [12]. Uterotonics (oxytocin, prostaglandin E1/misoprostol, methylergonovine, prostaglandin 15-methyl F2α/carboprost tromethamine, and prostaglandin E2/dinoprostone) are commonly prescribed in PPH management. Of these, only oxytocin, methylergonovine, and carboprost tromethamine are approved by the FDA for PPH management; use of other medications is off-label [14,15]. Studies so far have focused on specific aspects including uterine balloon tamponade in resource-poor settings [16], emergency hysterectomy for PPH [17], anti-fibrinolytic agents [18], and pregnancy outcomes after surgical treatment [19]. Management of PPH varies in different resource settings; more expensive and/or heat-labile treatment options requiring refrigeration may be difficult or impossible to use in limited-resource settings like in Southeast Asia [20]. Social and cultural inequalities, along with issues concerning the accessibility and comprehensiveness of healthcare, further complicate the management of PPH in these countries.

Considering the increasing PPH burden in Southeast Asia, there is an unmet need to understand the current PPH treatment and management paradigms in these countries and have specific treatment and management plans to address PPH, especially in such limited-resource settings. The current scoping review thus focuses on understanding the patient journey, assessment criteria, current treatment landscape, and unmet needs for PPH and sPPH in Southeast Asia with a special focus on Malaysia and developing evidence-based as well as practice-based recommendations for the management of PPH in Malaysia. The review also highlights the evolving treatment landscape for PPH in Southeast Asia.

## Review

Methodology

This scoping review methodology was developed in accordance with the guidelines given by Levac et al. (2010) [21]. A panel of experts on the pharmacological treatment of PPH from Malaysia performed a detailed literature search of the available English-language medical literature using MEDLINE and Google Scholar (January 2013 to 11 January 2023) using Preferred Reporting Items for Systematic Reviews and Meta-analysis (PRISMA) reporting guidelines and provided specific evidence-based and experience-based clinical recommendations for effective management of PPH in Southeast Asia, especially Malaysia, in consideration with the current Malaysian guidelines.

Details of the comprehensive search and PICO (population, intervention, comparator, outcome) framework are provided in Table 1 and Table 2, respectively. Abstracts from potentially relevant publications were read for content, and only observational and/or clinical studies conducted in pregnant women (>16 years old) from Southeast Asia were included in this review article. Studies focused on identifying the risk factors for PPH or those describing the tools for diagnosing PPH were all excluded from the review. Reviews, conference abstracts, and grey literature were also excluded. Statistical analysis was not conducted as this scoping review aimed to map available evidence descriptively, following standard scoping review methodology. Results were presented descriptively.

**Table 1:** Search Terms Used for the Review

Search Engine	Search Strings
PubMed	management[tiab] OR therapy[tiab] OR “Therapeutics” OR treatment[tiab] OR “fundal massage”[tiab] OR “uterine massage”[tiab] OR ((fundus[tiab] OR fundal[tiab] OR uterus[tiab] OR “uterus”[MeSH Terms] OR uterine[tiab]) AND (massage[tiab] OR “massage”[MeSH Terms])) OR compression[tiab] OR “antishock garment”[tiab] OR “antishock garments”[tiab] OR “Gravity Suits”[MeSH Terms] OR “Fluid Therapy”[mh] OR uterotonic[tiab] OR oxytocin[tiab] OR “oxytocin”[MeSH Terms] OR Pitocin[tiab] OR oxytoxic[tiab] OR Oxytocics[mesh] OR misoprostol[tiab] OR “misoprostol”[MeSH Terms] OR Cytotec[tiab] OR methylergonovine[tiab] OR “methylergonovine”[MeSH Terms] OR methergine[tiab] OR ergonovine[tiab] OR “ergonovine”[MeSH Terms] OR ergotrate[tiab] OR carboprost[tiab] OR “carboprost”[MeSH Terms] OR “carboprost tromethamine”OR transfusion[tiab] OR “Blood Transfusion”[mh] OR “fluid resuscitation”[tiab] OR “isotonic crystalloids”[tiab] OR “isotonic crystalloid”[tiab] OROR fibrinogen[tiab] OR “fibrinogen”[MeSH Terms] OR “fresh frozen plasma”[tiab] OR “plasma”[MeSH Terms] OR “uterine tamponade”[tiab] OR “balloon tamponade”[tiab] OR “intrauterine balloon”[tiab] OR “uterine balloon”[tiab] OR “Uterine Balloon Tamponade”[mh] OR “Bakri balloon”[tiab] OR ((uterus[tiab] OR “uterus”[MeSH Terms] OR uterine[tiab] OR intrauterine[tiab]) OR “Bakri balloon”[tiab] OR “arterial embolization”[tiab] OR “artery embolization”[tiab] OR “artery ligation”[tiab] OR “ligation”[MeSH Terms] OR “arterial ligation”[tiab] OR “laceration repair”[tiab] OR “recombinant activated factor VII”[tiab] OR “rFVIIa”[tiab] OR “Factor VIIa”[mh] OR Laparotomy[tiab] OR “laparotomy”[MeSH Terms] OR Hysterectomy[tiab] OR “hysterectomy”[MeSH Terms] OR “B-lynch”[tiab] OR “Suture Techniques”[MeSH Terms] OR suture[tiab] OR suturing[tiab] OR “Uterine Inertia/prevention and control”[Mesh] OR “Uterine Inertia/therapy”[Mesh] OR “Uterine Inversion/therapy”[Mesh] OR “Uterine Rupture/therapy”[Mesh] OR “urinary catheterization”[tiab] OR “Urinary Catheterization”[Mesh] OR “catheter balloon”[tiab] OR “balloon catheter”[tiab] OR “foley catheter”[tiab] OR “condom catheter”[tiab] OR “condom tamponade”[tiab] OR (Condoms[Mesh] AND balloon[tiab]) OR “Rusch balloon”[tiab] OR AND placenta[tiab]) OR “Placenta, Retained/therapy”[Mesh] AND “adverse effects”[Subheading] OR unsafe[tiab] OR safety[tiab] OR harm[tiab] OR harms[tiab] OR harmful[tiab] OR complication[tiab] OR complications[tiab] OR “side-effect”[tiab] OR “side-effects”[tiab]. "Malaysia" OR Malaysian women OR Asia pacific OR Asian women OR postpartum hemorrhage”[MeSH Terms] OR “postpartum hemorrhage”[tiab] OR “postpartum haemorrhage”[tiab] OR (PPH[tiab] AND postpartum[tiab]) OR “obstetric hemorrhage”[tiab] OR “obstetric haemorrhage”[tiab] OR ((“postpartum period”[MeSH Terms] OR post-partum[tiab]) AND (“hemorrhage”[MeSH Terms] OR hemorrhage[tiab] OR haemorrhage[tiab]))

**Table 2:** PICO Framework Used in This Review ICU: Intensive care unit; PICO: Population, intervention, comparator, outcome

PICO	Description
Population	Women with postpartum hemorrhage immediately post-birth to 12 weeks postpartum following pregnancy >24 weeks' gestation.
Intervention	Compression techniques (external uterine massage, bimanual compression, aortic compression). Medications (oxytocin (Pitocin), misoprostol (Cytotec), methylergonovine maleate (Methergine), carboprost tromethamine (Hemabate), dinoprostone (Prostin E2), recombinant activated factor VIIa (NovoSeven), and tranexamic acid (Cyklokapron)). Devices (Bakri postpartum balloon, Foley catheter, Sengstaken-Blakemore tube, Rusch balloon). Procedures (manual removal of placenta, manual evacuation of clot, uterine balloon tamponade, uterine artery embolization, laceration repair).
Comparator	Different intervention (any intervention compared with any other intervention)
Outcome	Transfusion, ICU admission, Anaemia, Length of stay, Blood loss

Results

**Figure 2:**
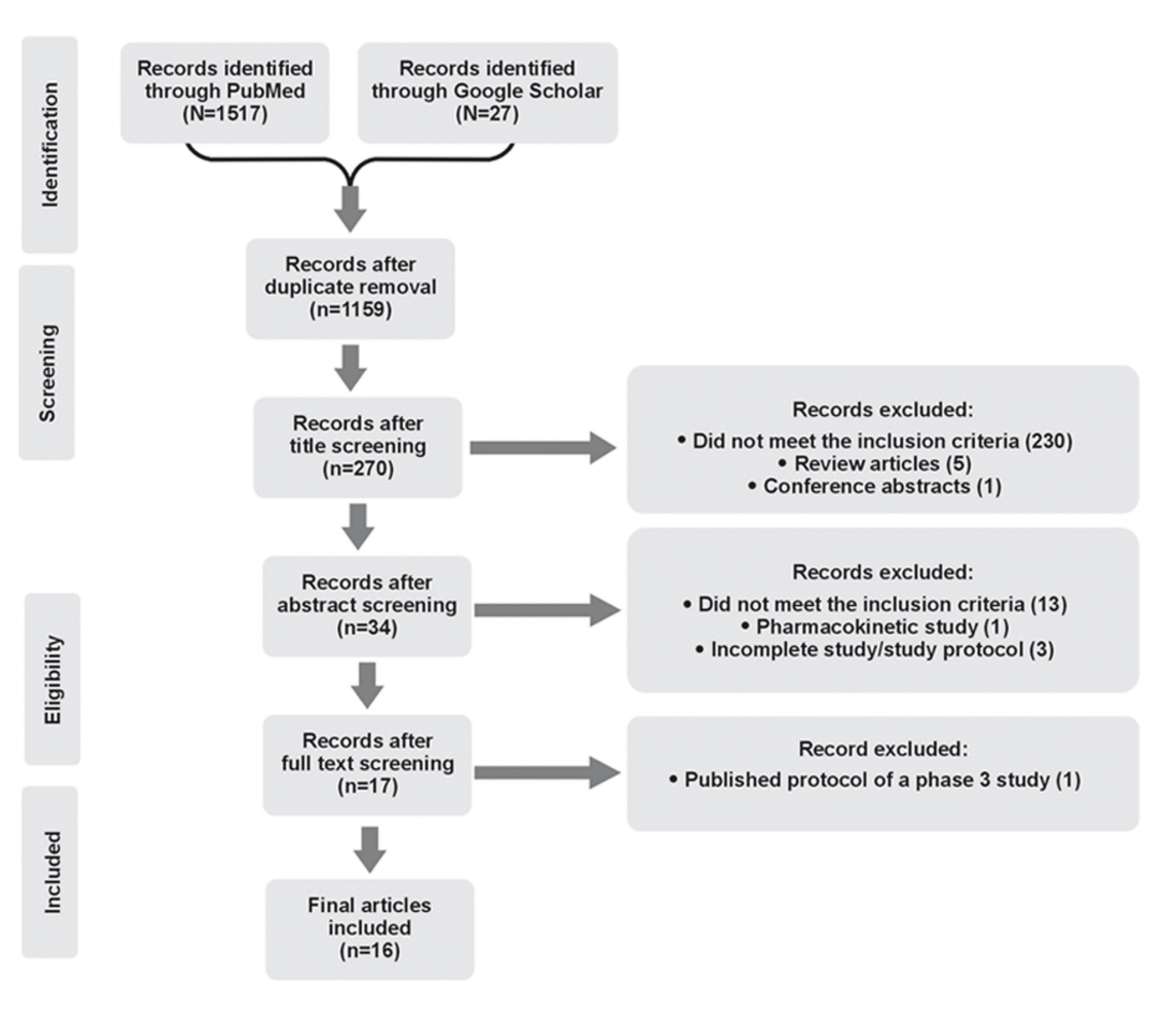
PRISMA Diagram for the Current Review Detailing the Strategy Used PRISMA: Preferred Reporting Items for Systematic Reviews and Meta-analysis

Figure 2 represents the PRISMA flow chart outlining details of the study identification and selection process. A total of 1544 articles were screened (PubMed: 1517, Google Scholar: 27); after screening for duplicates, 17 articles met the inclusion criteria and were selected for this review. Of these, one study was a published protocol of phase 3 study and hence was excluded from this review. The baseline characteristics of these studies are presented in Table 3 and details of these 16 studies are presented in Table 4. PPH is one area of medical education research that has not yet been systematically examined in Malaysia as was evident from the searches conducted for the last 10 years.

**Table 3:** Baseline Characteristics from the Articles Reviewed *Some studies included in this table were conducted simultaneously across multiple countries

Parameters	No. of studies, n (%)
Total number of studies included in this scoping review (N)	16 (100)
Type of delivery, N=16	
Vaginal delivery	7 (44)
Caesarean delivery	3 (19)
Not mentioned	6 (37)
Country/Region, N=17*	
Thailand	3 (18)
India	7 (41)
Hong kong	1 (6)
Nepal	2 (12)
Pakistan	4 (24)

**Table 4:** Summary of 16 Studies that Matched the Inclusion Criteria and Were Included in the Scoping Review DB: Double blind, DIC: Disseminated intravascular coagulation; IM: Intramuscular, IU: Intrauterine; IV: Intravenous, PPH: Postpartum haemorrhage, RCT: Randomized controlled trial, rFVIIa: Recombinant activated factor VIIa, sPPH: Severe post-partum haemorrhage; TB: Triple blind

S.No	Author	Year	Population	Population Size	Study Type	Country	Intervention	Comparator	Blood Loss	Method of Estimation of Blood Loss	Results
1	Khanum et al. [22]	2023	sPPH Vaginal delivery – 37%, Cesarean section – 63%	15	Observational study	Pakistan	rFVIIa	-	Median and mean blood loss were 8639 mL and 11835 mL, respectively	Not mentioned	rFVIIa is a useful tool in severe cases of PPH who are nonresponsive to standard therapy
2	Pradhan et al. [23]	2022	PPH due to uterine atony in whom medical treatment had failed. 73.3% were multigravida, and 63.2% had vaginal delivery	19	Retrospective study	Nepal	Condom tamponade	-	The average amount of blood loss was 1330 mL	Suction machine	Condom tamponade was successful in 94.7% women with uterine atony, who did not respond to uterotonic drugs, and 5.3% required peripartum hysterectomy
3	Mohta et al. [24]	2022	Healthy laboring patients with uncomplicated, singleton term pregnancy received oxytocin for induction or augmentation of labor, undergoing non-elective cesarean section under spinal anesthesia	105	Prospective DB RCT	India	oxytocin infusions at rates of 2.5 IU/h (Group 2.5), 5 IU/h (Group 5), or 10 IU/h (Group 10) following a 3 IU slow bolus.	Control	>500ml	Suction bottle, sponges, and drapes	Oxytocin infusions at 5 IU/h and 10 IU/h are more effective in reducing blood loss and preventing PPH than 2.5 IU/h.
4	Dawood et al. [25]	2022	Primary PPH caused by uterine atony	118	Retrospective	Pakistan	Bakri balloon	-	Mean blood loss was 1208.5±227.9 ml.	Tray	Efficacy of balloon tamponade was found in 108 (91.5%) cases, and 10 (8.5%) cases were non-effective
5	Akhtar et al. [26]	2022	PPH due to uterine atony, which failed to respond to medical treatment, vaginal delivery	83	Monocentric retrospective impact study	Pakistan	Foley’s catheter balloon tamponade	-	Not mentioned	Not mentioned	Uterine tamponade balloon to manage PPH for the uterine atony in cases where the medical treatment failed, and balloon tamponade was effective in controlling PPH in 78 (93.98%) cases. Only 5 (6.02%) patients needed further surgical intervention
6	Patil et al. [27]	2021	Atonic PPH Vaginal delivery – 8, Caesarean delivery - 42	50	Retrospective cohort study	India	Bakri balloon	-	-		Intrauterine balloon tamponade using the Bakri balloon is effective for the control of atonic PPH in the majority of cases.
7	Yu et al. [28]	2020	Diagnosed with placenta previa at 34 weeks and required cesarean delivery	40	Prospective RCT	Hong Kong	Internal iliac artery balloon catheter placement was performed before cesarean delivery, and then balloon inflation after delivery	Standard Control	1451 (1024-2388) mL vs 1454 (888-2300) mL – median intraoperative	Disposable delivery drape with a blood collection system applied around the abdominal wound for accurate collection and measurement	Prophylactic internal iliac artery balloon occlusion in placenta previa patients undergoing cesarean delivery did not reduce PPH
8	Akhtar et al. [29]	2020	Primary PPH after vaginal delivery	80	Experimental study	Pakistan	Balloon tamponade insertion of Sengstaken Blakemore oesophageal catheter	-	Mean estimated blood loss 1125+320 ml,	Pre-weighed pads	Nine cases where balloon tamponade did not show efficacy, five (55.6%) had incorrect placement due to the large size of the uterus, whereas two (22.2%) women had a fibroid uterus, and two (22.2%) developed disseminated intravascular coagulation (DIC).
9	Yadav et al. [30]	2019	Vaginal delivery	200	Prospective randomized comparative study	India	Condom catheter	800mh Misoprostol	Mean - 663.80ml	Not mentioned	Balloon catheter is better than misoprostol for controlling PPH.
10	Amornpetchakul et al. [31]	2018	At least one risk factor for atonic postpartum hemorrhage, after vaginal delivery	350	TB RCT	Thailand	100 mcg carbetocin	5 U oxytocin		Postpartum drape with calibrated bag	The carbetocin group had less postpartum blood loss, a lower incidence of atonic PPH, less usage of additional uterotonic drugs, and a lower incidence of postpartum anemia
11	Widmer et al. [32]	2018	Vaginal birth	29645	DB RT noninferiority	Argentina, Egypt, India, Kenya, Nigeria, Singapore, South Africa, Thailand, Uganda, and the United Kingdom	IM carbetocin100 μg	Oxytocin 10 IU	≥500ml	Plastic drape for blood collection (BRASSS-V Drape) was placed under the woman’s buttocks.	IM 100 μg of heat-stable carbetocin was noninferior to 10 IU of oxytocin for the prevention of PPH in ≥500ml blood loss cases. Noninferiority was not shown for the outcome of blood loss of at least 1000 ml.
12	Cecilia et al. [33]	2018	Elective emergency cesarian delivery	271 Group A (oxytoci n= 10 units; n=135) and Group B (oxytoci n= 30 units; n= 136)	DB RCT	India	IV 10 units oxytocin in 500 ml over 2–4 h	IV30 units oxytocin over 8–12 h	Total mean estimated blood loss was 400 ml in both the groups (P= 0.44)	Mops	A low dose of oxytocin might be able to achieve the same degree of uterine contraction but with fewer side effects than a high dose of oxytocin.
13	Joshi [34]	2017	PPH unresponsive to pharmaceutical agents	29	Retrospective descriptive study	Nepal	Intrauterine condom tamponade - Foleys catheter	-	500 to 4200ml	Not mentioned	Prophylactic use may aid in reducing blood loss and morbidity and preserving productive capacity
14	Humza et al. [35]	2017	Pregnant women	120		Pakistan	Syntometrin IV, + mesoprostol 1000mcg and syntocinon 10 units, if this failed, b lynch suture, massage, internal iliac artery ligation	-	Not mentioned	Not mentioned	Effective management of PPH
15	Widmer et al. [36]	2016	Vaginal delivery	30000	RCT	Argentina, Egypt, India, Kenya, Nigeria, Singapore, South Africa, Thailand, Uganda, and the United Kingdom	IM 100 μg Carbetocin	IM 10 IU oxytocin	Not mentioned	Calibrated drape	Carbetocin RTS dose of 100 μg IM will be efficacious and have a safety profile that is equal to or better than the well-characterized safety profile of 100 μg IV when used in women following caesarean section.
16	Priya et al. [37]	2015	Low-risk women, singleton pregnancy, vaginal delivery	500 group 1 (misoprostol) and group 2 (oxytocin)	RCT	India	400 mcg misoprostol sublingually	IM 10 units oxytocin	≥500ml	Flat bowl kept under buttocks after the delivery of the placenta	Women in the oxytocin group had more blood loss compared to misoprostol group.

Awareness of PPH and sPPH Among Women and Healthcare Professionals (HCPs) and the Burden of Disease in Malaysia

In Malaysia, PPH represents a critical public health challenge, accounting for 26% of maternal morbidity in 2017 according to the Ministry of Health Malaysia [38], with the national maternal mortality ratio rising to 24.8/100,000 live births by 2020. PPH was identified as the leading cause of maternal mortality in 2019 and remained among the top five causes in 2020 [23,39]. Regional variations within Malaysia are evident, with Selangor reporting a particularly high maternal mortality ratio of 22/100,000 live births in 2014 and 184 postpartum maternal deaths across nine districts from 2013 to 2019, [40] knowledge, awareness, and attitude about PPH amongst women in Malaysia.

In a survey conducted among Malay mothers in Kuantan, Pahang, Malaysia, only 50% of the respondents were aware of the signs and symptoms of PPH and about 77% sought help from traditional healers to relieve their PPH symptoms rather than visiting healthcare facilities [39]. A study conducted in Kelantan, Malaysia, documented a case where the woman preferred home delivery due to fear of hospitals, particularly needles while another woman delayed seeking help for about six hours despite severe bleeding due to her preference for family presence and aversion to hospital mortality [11]. Such cases highlight the women’s predisposition to seek healthcare and an increased need for creating awareness to change their perception of the quality of care.

In low- and middle-income countries, it was observed that ICU admission with obstetric complications corresponded to 3.1 per 1000 live births with the majority of obstetric morbidities transferred to ICU related to hysterectomy (89.5%) and haemorrhage due to placental implantation disorders (76.9 %) [41,42]. Approximately 20% to 50% of women with maternal near-misses failed to receive ICU care from events like PPH. Approximately half of the women with severe maternal morbidity (46.3%) and maternal near-miss (55.3%) developed complications within 12 hours of admission, of which 68.3% and 61.5 % respectively were referred cases, indicative of the delay in the referral system [41,42]. These observations indicate incomplete awareness among HCPs in these countries leading to failure in accessing tertiary centres and/or referral systems. In one study conducted in South Ethiopia, the knowledge and practice of HCPs with regard to the prevention of PPH by actively managing the third stage of labour were unsatisfactory, with only 37.7% of the health care providers in obstetrics having enough knowledge on how to manage the third stage of labour during the childbearing period [43]. Another important aspect was the limited awareness of the HCPs towards prompt referral of women with PPH [44,45].

Malaysian healthcare providers face significant PPH management gaps despite government standardization efforts through Ministry of Health training manuals and reference guides [1,2]. System constraints include workforce limitations, urban-rural resource disparities, and care escalation inefficiencies, particularly at primary and district levels [46,47]. Key knowledge deficits involve poor blood loss identification, inaccurate estimation methods, delayed surgical escalation, and lack of confidence with newer interventions (tranexamic acid, balloon tamponade, advanced surgical techniques). While trainees possess moderate PPH knowledge, they struggle with practical application, especially emergency responses and care bundle implementation [39,46,47].

High in-hospital mortality rates suggest that suboptimal care quality requires review concerning clinical PPH management [42]. Apart from the use of oxytocin for its prevention and treatment, other aspects of care such as shock management, haemostatic interventions, an adequate donated blood supply and prompt surgical interventions are essential. Midwives, nurses and doctors often find it difficult to conduct genital tract examinations due to discomfort or reluctance or cultural beliefs of women in Southeast Asia [24,28,32,36]. Limited awareness or access to current PPH treatment guidelines, limited resources and understaffing hinder prompt response and treatment [37].

Current training programs consist of mandatory hospital-based in-service courses and the use of national training manuals, which are provided to both undergraduate and postgraduate trainees. These programs aim to educate healthcare workers in both public and private sectors, with core objectives such as standardizing approaches across various facility levels and improving referral pathways [46]. Clearly, there is a continuing education need for more frequent, simulation-based refresher courses, given that evidence shows hands-on simulation training yields significant, lasting improvements in knowledge and clinical performance related to PPH management [48].

Challenges Associated With Diagnosis of PPH and sPPH in Malaysia

Differential diagnostic criteria for blood loss in a real-world setting: The Malaysia MOH guidelines mention secondary PPH as abnormal or heavy bleeding that occurs between 24 hours to six weeks post-delivery [49]. Common clinical presentations of secondary PPH include sudden and very heavy vaginal bleeding, increasing vaginal bleeding, passing clots or placental tissue or membranes, strong or unpleasant odour of vaginal bleeding or discharge indicating an infection, or other symptoms of infection such as fever, chills, abdominal pain, headache or muscle aches [50].

However, in real-world practice, clinicians use varying definitions of PPH especially those for sPPH, which are often based on inaccurate estimates of blood loss, thereby making it difficult to identify sPPH. This was evident in the current study where only three out of 17 publications used a blood loss of ≥500 mL to define PPH [20,24,36]. All three publications involved women who were deemed for normal vaginal delivery. In one study, women undergoing caesarean section with a blood loss of 400 mL were deemed eligible for administration of uterotonics [37]. Another study by Yadav et al. mentioned a mean blood loss of 663 mL as PPH [30]. Five studies classified women with blood loss above 1000 mL as primary PPH [23,25,28,29,34]. One study from Pakistan conducted in 15 pregnant women with sPPH reported a mean blood loss of 11835 mL [22]. This is an alarming observation denoting the lack of proper assessment criteria in defining sPPH coupled with the lack of proper measurement of blood loss in the country. One study by Yu et al. involving pregnant women with placenta previa demonstrated a median blood loss of approximately 1400 mL [28]. These studies suggest that there is an unmet need to create adequate awareness among practising clinicians to identify the signs and symptoms of PPH with early and timely diagnosis.

Lack of appropriate methods for estimating blood loss in Southeast Asia: Visual estimation of blood loss at birth is a major part of PPH diagnosis. However, the lack of standard methods to measure the blood loss in these women, as seen in the current review, results in underestimation of blood loss by the caregivers and doctors thereby delaying the diagnosis of PPH. The most common method of blood loss estimation involved postpartum drapes [24,28,32,36], followed by preweighed pads/mops/tray [24,25,29,31,33,37]. Both these methods of measuring blood loss have limitations; it is difficult to measure the immediate blood pooling around the patient in case of drapes and trays/mops/pads as they do not allow real-time monitoring of blood loss [51]. The visual estimation of blood loss is an inaccurate method which frequently causes underestimation of blood loss resulting in delay of treatment. There is a need for a more accurate assessment tool for blood loss estimation which is vital in improving the clinical landscape of PPH and sPPH in Southeast Asia.

Patient intrinsic factors for PPH and sPPH: Every pregnancy is at risk of developing PPH [52]. Another important challenge in identifying PPH and sPPH adequately is patient intrinsic factors like uterine atony. In fact, uterine atony is the leading cause of PPH resulting in serious consequences like severe anaemia, shock, and death especially in African and some Southeast Asian countries where access to quality health care services is not feasible due to financial constraints [37,53]. In Malaysia, approximately 50% of maternal deaths due to PPH have been attributed to uterine atony [6]. In another retrospective study from Malaysia, it was observed that the leading causes of PPH were retained placenta, followed by uterine atony, uterine rupture and placenta accreta [54]. Placenta accreta has been reported to be one of the major causes of PPH in Malaysia followed by placenta increta and percreta [55], also referred to as the morbidly adherent placenta spectrum (MAPS). MAPS is categorised as acrefair if the depth of placenta invasion is mild and percreta when it is severe. In Malaysia, grand multiparity is common which is also a risk factor for PPH (secondary uterine atony) [56]. About 30% to 40% of PPH-related mortality in Malaysia occurred in patients with a parity of more than five [56]. Risk factors that are significantly (p<0.01) associated with PPH in Malaysia include age, anaemia during the antenatal period, uterine fibroid, prolonged labour (>8 hours), instrumental delivery, extensive vaginal wall tear, cervical tear, third and fourth-degree tear, uterine atony and vascular lower segment during caesarean section [57]. Additionally, Southeast Asian ethnicity is significantly associated with an increased risk of haemorrhage [58]. This could be due to genetic makeup such as a defect in coagulation or tissue elasticity. Lack of adequate iron-folate supplementation, lack of access to a nutritious diet, and antenatal risk factors are a cause of concern due to the lack of awareness and education among the mothers in these countries [27].

In the current scoping review, six studies reported PPH in pregnant women with uterine atony [23,25-28,31]. Of these, only two studies mentioned the mean blood loss of 1208 mL and 1330 mL in these women [23,25]. There is thus an unmet need to develop concrete guidelines focussed on diagnosing PPH and sPPH using better assessment criteria apart from the volume of blood loss and consider all risk factors to identify candidates who may be prone to the development of PPH and sPPH (Table 5). Furthermore, although the most common etiology of PPH is uterine atony occurring in about 80% of cases, the majority of women who develop PPH have no identifiable risk factors. The Malaysia MOH guidelines suggest that in such cases, factors associated with uterine atony, such as multiple gestation, polyhydramnios, high parity, and prolonged labour, should be identified along with other factors like retained placenta or clots, lacerations, uterine rupture or inversion, and inherited or acquired coagulation abnormalities (Table 6). It is also essential to note that blood loss in a few patients may be concealed within genital tract haematomas or intraabdominal collections and hence not visible. According to the Malaysia MOH guidelines, active management of the third stage of labour (prophylactic uterotonic administration, early cord clamping and controlled cord traction for placental delivery) is critical for the prevention and treatment of PPH.

**Table 5:** Management of PPH and sPPH Considering Different Risk Factors* *Based on MOH Malaysia guidelines for prophylaxis and treatment plan for PPH. Table created by the authors based on information from [46] ABG: Arterial blood gas; Ca/Ca+2: Calcium; CCT: Controlled cord traction; Coags: Coagulation profile; EUA: Emergency use authorisations; FBC: Full blood count; FFP: Fresh frozen plasma; IM: Intramuscular; IV: Intravenous; LFT: Liver function test; MRP: Maternal recognition of pregnancy; PPH: Postpartum haemorrhage; sPPH: Severe postpartum haemorrhage; PR: Per rectal; RBC: Red blood cells, 4Ts: Tone, Trauma, Thrombin, Tissue

Risk Factors and Causes	Directed Therapy	Surgical Interventions, If Directed Therapy Does Not Stop Bleeding
PPH		
Tissue-related: Retained placenta	Ensure third-stage uterotonics are given. Apply CCT & attempt delivery: - If it is successful: Check the placenta is complete. If unsuccessful/missing cotyledon or membranes: For MRP/digital evacuation (preferably in OT) with prophylactic broad-spectrum antibiotics. Post-evacuation: Massage uterus, assess tone, and give prophylactic oxytocin infusion	Manual removal with or without curettage
Tone-related: Uterine atony	Massage the uterus. Ensure third-stage oxytocin is given. Expel any blood clots. Ensure bladder is empty. Administer IV drug (uterotonics): - IV oxytocin 5IU slowly, then oxytocin infusion - IM Syntometrine 1 ampoule - IM Carborprost 250mcg - PR Misoprostol	Intrauterine balloon tamponade. Angiographic embolisation. Laparotomy: B-Lynch suture, pelvic devascularisation, and Hysterectomy (consider early)
Trauma: Genital tract trauma	Massage uterus. Ensure third-stage oxytocin given. Expel any blood clots; ensure bladder empty. Administer IV drug (uterotonics): IV oxytocin 5IU slowly, then oxytocin infusion, IM Syntometrine 1 ampoule, IM Carborprost 250mcg, and PR Misoprostol	Optimise exposure with retractors. Inspect cervix, vagina, and perineum. Assess uterus intact. Repair: secure apex
Thrombin: Coagulopathy	Send FBC, Coags, LFTs, ABG. Do not wait for blood results to treat. Give RBC, FFP, and platelets, Cryoprecipitate if fibrinogen <2.5g/dL, Ca Gluconate if Ca^+2^ <1.1mmol/L. Avoid hypothermia and acidosis	Pelvic devascularisation. Hysterectomy (consider early)
Other causes	Look for other causes (not immediately obvious earlier). Consider Uterine rupture, Uterine inversion (irregular fundus), Puerperal haematoma, non-genital cause (e.g. amniotic fluid embolism, subcapsular liver rupture). Repeat assessment of the 4 T’s	EUA. Laparotomy
sPPH		
Tone: Subinvolution of uterus	Uterine massage. Bimanual compression. Uterotonic drugs	Local control. Bimanual compression. Ballon tamponade. Uterine/vaginal packing before definitive surgery. Selective arterial embolisation (if available). Surgery (Locate source, stem bleeding). EUA, repair laceration. Pelvic devascularisation. Hysterectomy
Tissue: Retained placenta, infection	If complicated with atonic uterus, see under ‘Tone’. Antibiotics. Evacuation of retained placenta	
Trauma: Missed vaginal lacerations, haematomas	Uterine massage. Bimanual compression. Uterotonic drugs	
Thrombin Coagulopathy, anticoagulants	Replace factors. Reverse anticoagulation	

**Table 6:** Expert Recommendations for Management of PPH in Malaysia Note: Recommendations presented in this table are based on expert professional experience and specialized knowledge in the relevant domain. Table created by the authors using [46]. MOH: Ministry of health; MTP: Medical termination of pregnancy; PPH: Postpartum haemorrhage; rFVIIa: Recombinant Factor VIIa; sPPH: Severe postpartum haemorrhage; VTE: Venous thromboembolism

Conditions	Recommendations
Diagnostic recommendations for PPH and sPPH	
Blood loss limits for PPH and sPPH	Follow the MOH definition for primary PPH (blood loss of 500 mL or more from the genital tract within 24 hours of birth of a baby, regardless of method of delivery) and secondary PPH (blood loss of 500 mL or more from the genital tract after 24 hours to 6 weeks post-delivery). Blood loss of 1.5 L or more within 24 hours after giving birth, regardless of method of delivery, should be considered as sPPH. Amount of blood loss should not be the only determining factor for sPPH. Additional factors similar to the venous thromboembolism risk scoring system should be considered for sPPH diagnosis.
Additional factors to be considered for sPPH diagnosis	sPPH diagnosis should be extended to any patient with hypovolaemic shock regardless of the amount of estimated blood loss within 24 hours after giving birth, especially in anaemic patients. Patients with underlying medical disorders may develop complications earlier than patients with no comorbidities. So, clinical assessment, such as checking the vital signs, is essential when evaluating patients with sPPH. Consider adding circulatory parameters, such as heart rate and blood pressure, as a vital evaluation component for sPPH.
Signs and symptoms of sPPH that should trigger attention	Feeling thirsty, dizziness, lethargy or shortness of breath should trigger the attending doctor to call for more attention to the patient Persistent blood loss even after the uterus has adequately contracted after delivery is also a cause for concern and should trigger a call for more attention to the patient Patient exhibiting any clinical signs of haemodynamic instability should be a cause for concern for the attending doctor. Patients with PPH and sPPH should be monitored closely in the High Dependency Unit (HDU), Post-anaesthesia Care Unit (PACU) or Intensive Care Unit (ICU) for at least 12–24 hours or until the patient stabilises haemodynamically.
	Application of the Obstetric Shock Index could be useful in the decision-making process when treating PPH patients, in which case, whenever the patient’s heart rate is more than the systolic blood pressure, a call for help should be issued to get more attention to the patient.
Managing PPH disease progression	Patients with risk factors such as grand multiparity and multiple pregnancy should be monitored closely, as PPH progression in such patients is faster than in those without risk factors. Obtain symptoms for cases of PPH where there is no visible bleeding, such as in uterine rupture.
Estimation of blood loss	Weighing the blood-soaked gauze improves the accuracy of blood loss estimation. This would require weight-measuring devices to be readily available in the labour rooms and wards. Evaluate more accurate methods of estimating blood loss.
Recommendations for treatment management of PPH	
PPH in vaginal delivery	First-line treatment should be oxytocin and tranexamic acid Second-line treatment can be syntometrine or misoprostol In case of prolonged bleeding due to uterine atony, a uterine tamponade balloon can be applied If prolonged bleeding is due to retained placenta, proceed with the removal of the placenta For genital tract trauma cases, suturing should be done at the site of the trauma Internal iliac artery ligation or hysterectomy should be considered if the bleeding persists after each procedure.
PPH in caesarean delivery	First-line treatment should be oxytocin along with tranexamic acid. Second-line treatment can be syntometrine or carboprost. In case of prolonged bleeding due to uterine atony, a uterine tamponade balloon such as the Bakri balloon, followed by the B-Lynch suture, may be considered. Hysterectomy should be considered as the last option if everything else fails.
Carbetocin use in managing PPH in caesarean delivery	Carbetocin may be used as first-line prophylaxis for patients with a high risk of developing PPH. Treatment with carbetocin should be used selectively, as it costs more than oxytocin.
MTP protocol in sPPH management	The MTP should include a ratio of 1:1:1 for packed cells, fresh frozen plasma, and cryoprecipitate, with the 1st cycle typically consisting of 4 units each. If haemostasis is not achieved after the first cycle, the second cycle of the MTP can be initiated. For ongoing bleeding, repeat blood investigations such as full blood count and coagulation profile every 30 minutes.
Dealing with placenta previa	Appropriate planning should be done to arrange for elective Caesarean section deliveries for patients with placenta praevia to minimise blood loss during the procedure by implementing adjustments such as making the surgical incision higher up on the abdomen so that the placenta is not affected during the surgery MTP can be activated for patients with placenta praevia which means the blood bank is informed in advance about the possibility of activating the MTP during or after the surgery. Constantly thawing the blood products such as the fresh frozen plasma for faster blood transfusion.
Decision on surgical interventions	Surgical intervention should be considered immediately once the medical treatment does not arrest the blood loss. Haemostatic agents should be given along with the uterotonics, which may reduce the need for surgical interventions.
Haemostatic treatments	Haemostatic treatment with tranexamic acid, fibrinogen and rFVIIa should be done in parallel to induce haemostasis. For the treatment of coagulopathy, it is vital to initiate treatment without waiting for the coagulation profile laboratory results. Low level of fibrinogen should alert the attending doctor that the patient might progress to sPPH. Administer tranexamic acid as soon as the diagnosis of PPH is made to increase the effectiveness of tranexamic acid Do not exceed the dose of tranexamic acid beyond 2 g to avoid renal complications.
rfVIIa treatment	The most ideal timing for the administration of rFVIIa should be at the beginning of PPH progression, not at the end It is also important to have a platelet count above 50 × 10^9^/L and a fibrinogen count above 2 g/L to maximise the effectiveness of the rFVIIa. The recommended dosage for rFVIIa should be 60 μg/kg which can be repeated once 30–60 minutes after the first dose.
Inclusion of rfVIIa in treatment protocol for sPPH and/or MTP	rFVIIa is suitable for reducing blood loss during an invasive procedure when it is administered right before the procedure, as it is effective within 10 minutes and lasts for 2–3 hours. Development of a treatment protocol specifically for sPPH that incorporates the use of rFVIIa or the inclusion of rFVIIa in the MTP would increase its accessibility
Ideal patients for rfVIIa treatment	Patients with sPPH regardless of the cause. The administration of rFVIIa is done concurrently with the definitive treatment of the underlying cause of the haemorrhage. Patients who are haemodynamically unstable due to blood loss, even after the 1st and 2nd lines of treatment have been given. Patients who have recalcitrant uterine atony, Patients who need to undergo surgical treatment for sPPH, and patients with sPPH who need to be transferred from smaller hospitals to specialist hospitals

Management of PPH and sPPH in Malaysia

**Figure 3:**
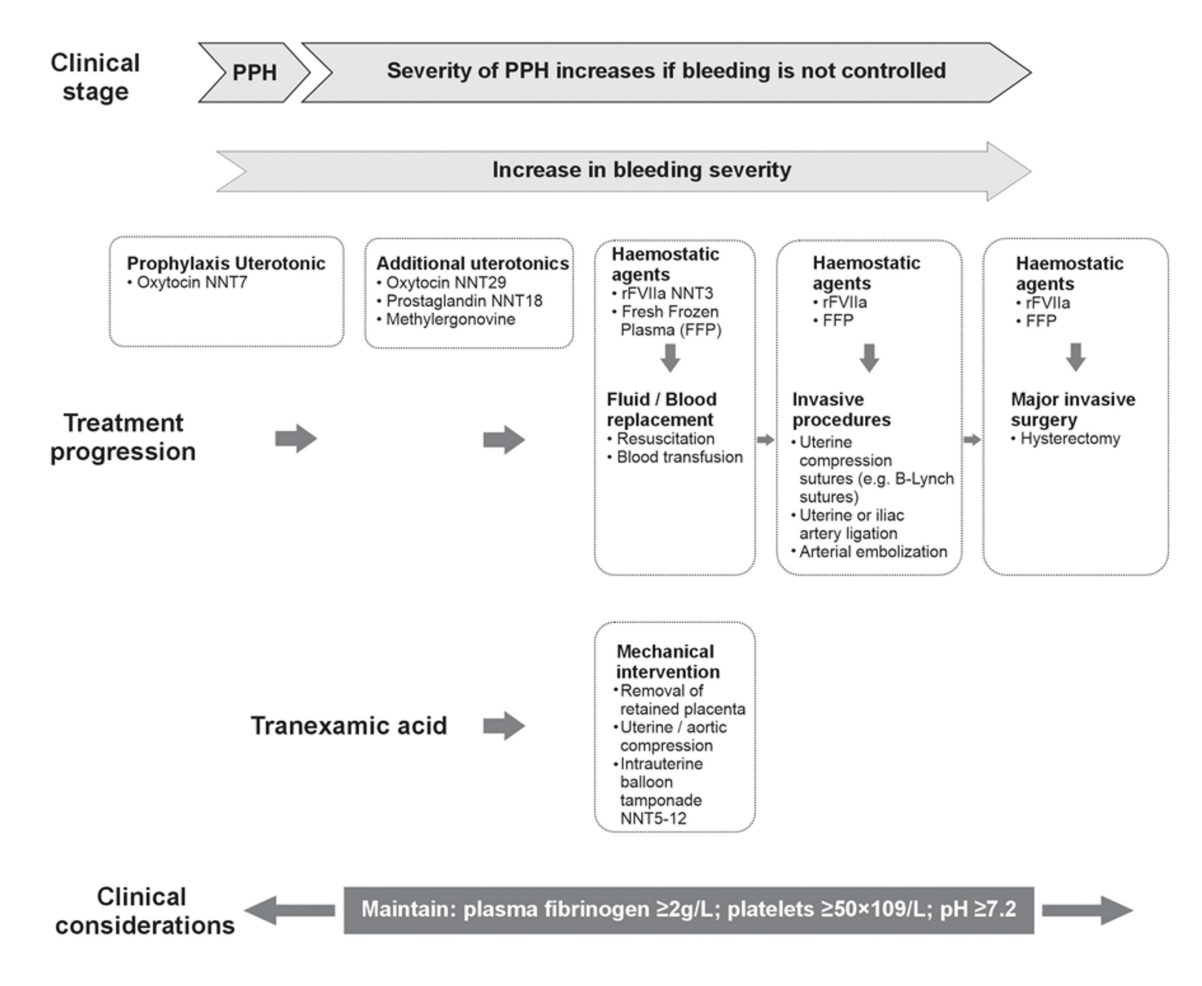
Interventions for PPH Management in Increasing Order of Bleeding Severity Figure created by the authors based on information from [14,15,42]

Challenges in PPH prophylaxis and first-line treatment management: Treatment of PPH depends on the etiology of PPH; the first line of treatment in pregnant women with uterine atony involves uterotonic agents such as oxytocin, misoprostol, and prostaglandin followed by invasive therapies and surgery as a last remedy (Figure 3).

This scoping review suggests that there have been mixed responses to different first-line treatments for PPH (oxytocin, carbetocin, syntometrin and misoprostol) in clinical practice. In one study, prophylactic treatment with sublingual misoprostol (400 µg) in the active management of the third stage of labour and PPH in 500 low-risk women undergoing vaginal delivery, was more effective in controlling PPH than intramuscular oxytocin administration (10 IU/hour) [37]. Carbetocin (100 µg) had similar efficacy to oxytocin (10 IU/hour) in treating PPH in vaginal delivery, however, it was not as effective in treating sPPH [36]. Intravenous carbetocin (100 µg) was found to be more effective in reducing atonic PPH than intravenous oxytocin (5 IU/hour) [31]. The study also demonstrated that the carbetocin-treated patients had a lower incidence of postpartum anaemia than oxytocin-treated. Considering the side effects associated with oxytocin use, two studies reported that lower doses of oxytocin (5-10 IU/hour) were as effective as higher doses (10-30 IU/hour) in patients undergoing elective or non-elective caesarean delivery, thereby bringing down the high dose-associated side effects in these patients [24,33]. However, although oxytocin and carbetocin are both recommended as prophylactic agents in treating PPH, their use is limited mostly due to their stability issues and refrigeration requirements which may not be feasible in many of the remote regions of Southeast Asian countries [31,37].

Management of Uterine Atony-Associated PPH in Southeast Asia

In the current scoping review, the use of IUBT was the most commonly used method to manage PPH in patients with uterine atony [23,25,26,28-30,34]. Most studies used IUBT in treating PPH due to the non-responsiveness of these patients to first-line treatments [23,26,29]. One study by Yadav et al. demonstrated that a condom catheter was more effective in controlling PPH in vaginal delivery than first-line treatment with misoprostol (800 µg) [30]. However, prophylactic IUBT occlusion in patients with placenta previa undergoing caesarean delivery did not reduce the PPH [28]. These studies indicate that there is a need for specialised equipments and skilled interventional radiologists along with discrete guidelines for selecting the right treatment regimen for different patient conditions.

Management of Disseminated Intravascular Coagulopathy-Associated PPH in Southeast Asia

**Figure 4:**
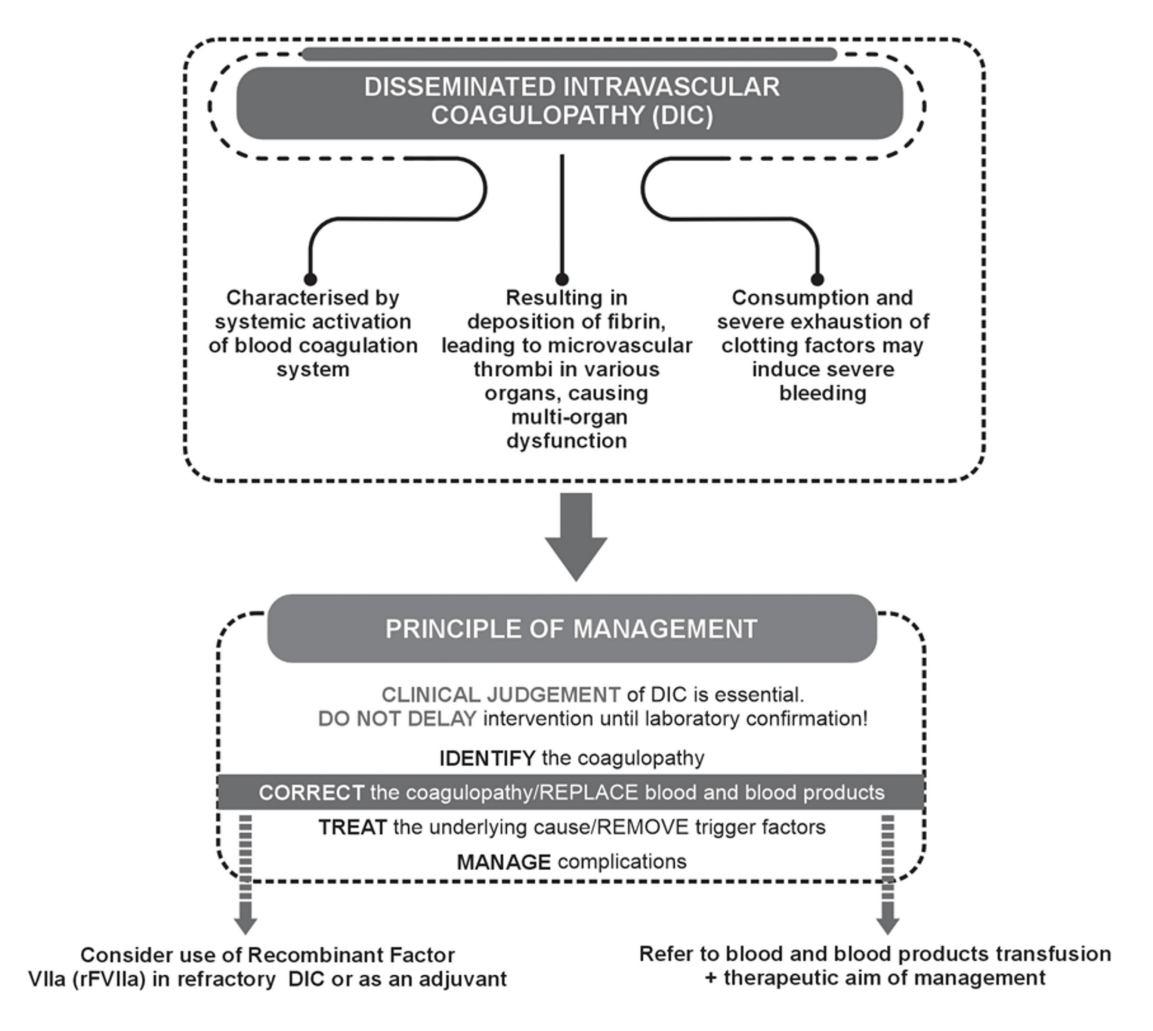
Strategies Aimed at the Inhibition of Coagulation Activation Based on the Pathogenesis of Microvascular Failure and Coagulation Activation in DIC Figure created by the authors based on information from [42,57] DIC: Disseminated intravascular coagulation

Obstetrical haemorrhage and especially disseminated intravascular coagulation (DIC) is a leading cause of maternal mortality worldwide [59]. DIC is always secondary to an underlying disorder. Indeed, it is associated with pregnancy complications such as placental abruption, HELLP (haemolysis, elevated liver enzymes, low platelet count) syndrome, preeclampsia, retained stillbirth, sepsis, PPH, acute fatty liver, and amniotic fluid embolism. Based on the pathogenesis of microvascular failure and coagulation activation in DIC, strategies aimed at the inhibition of coagulation activation have been found favourable in experimental and clinical studies (Figure 4). Limitations in the availability of emergency blood products such as fresh frozen plasma and platelets availability may lead to the worsening of DIC [30]. Most studies have been carried out in patients with sepsis, and evidence in obstetric DIC is limited. Tranexamic acid is used as an adjunct to blood product administration and has been studied in PPH. Recombinant activated factor VII (rFVIIa) is used as a second-line treatment in women when a massive transfusion does not halt blood loss [42]. All other treatment modalities are considered experimental at this stage (Figure 4).

The World Maternal Antifibrinolytic (WOMAN) study which was conducted in 21 countries (including Pakistan, Nepal, and Bangladesh) demonstrated that tranexamic acid (100 mg/mL) was effective in reducing maternal mortality due to PPH with no adverse effects [39]. However, the lack of ready availability and non-feasibility of intravenous injections of tranexamic acid at home or in settings in the region impacts its use in Southeast Asia. Many women in Southeast Asia are anaemic and the availability of blood for transfusion is limited; in such cases, a hysterectomy is implemented as an early intervention to prevent death from exsanguination [60].

Discussion

Our results, supported by Malaysia's maternal mortality ratio of 24.8/100,000 live births (2020) with PPH as the leading cause in 2019, suggest substantial PPH and sPPH burden in Southeast Asia, particularly Malaysia; however, significant healthcare provider knowledge gaps and low maternal awareness (50% symptom recognition, 77% preferring traditional healers) indicate potential under-recognition requiring further epidemiological investigation. Moreover, studies focusing on the treatment and management of PPH are very few, mostly in the form of case studies. There is a severe underestimation of both PPH and sPPH reported in studies from Southeast Asia. Literature on PPH and sPPH incidence in Malaysia is very limited. The current scoping review also demonstrated a lack of uniformity in the treatment management of PPH and sPPH. While there were very few studies demonstrating the use of oxytocin and carbetocin in resource-limited settings of Southeast Asia, the majority of the studies relied on procedures like IUBT, and internal iliac artery balloon to counter PPH (Table 3). There is thus a clear requirement to define the treatment management plan for both PPH and sPPH especially in low-resource countries.

Expert Recommendations for PPH and sPPH Diagnosis in Vaginal Delivery, Caesarean Delivery, and Other Comorbidities

Based on our practice experience in obstetrics and gynaecology over the years, and evidence from other countries where sPPH is managed effectively, blood loss of 1.5 L or more within 24 hours after giving birth regardless of method of delivery should be considered as sPPH (Table 6). Diagnosis of sPPH should be extended to any patient with hypovolaemic shock regardless of the amount of estimated blood loss within 24 hours after giving birth. For example, patients with underlying anaemia may develop hypovolaemic shock with an estimated blood loss of only 1 L. Hence, in such cases, the patient would warrant a diagnosis of sPPH based on the clinical signs and symptoms, regardless of the estimated blood loss. Furthermore, considering that Malaysia MOH’s criteria for hospitals to not exceed the sPPH occurrence by 0.75% of deliveries in 2023, the amount of estimated blood loss should not be the only determining factor in the management of sPPH and factors like the venous thromboembolism risk scoring system should be considered to determine if thromboprophylaxis is required (Table 5). Patients with underlying medical disorders may develop complications earlier than those with no comorbidities. It is thus essential to evaluate these patients using additional circulatory parameters, such as heart rate and blood pressure.

Any patient exhibiting clinical signs of haemodynamic instability should be a cause for concern for the attending doctor and if the patient’s condition does not improve after the first-line treatment, it should trigger a call for more attention to the patient. Symptoms such as excessive thirst, dizziness, lethargy, or shortness of breath should trigger the attending HCP, nurse or midwife to call for more attention to the patient. Besides that, persistent blood loss even after the uterus has adequately contracted post-delivery should also be of concern. PPH following vaginal deliveries or instrumental deliveries are more difficult to manage than caesarean deliveries and such patients are candidates for sPPH; such cases should be monitored closely for at least 12-24 hours or until the patient stabilises haemodynamically. Application of the Obstetric Shock Index could be useful in the decision-making process, especially in patients with increased heart rates. Risk factors such as grand multiparity and multiple pregnancy, should be considered in managing PPH, as the tendency of such patients to progress to sPPH is higher than those with no risk factors. It is also essential to monitor the symptoms closely, particularly for PPH cases where there is no visible bleeding such as in uterine rupture.

Of all the blood loss estimation methods currently used in Southeast Asia, weighing the blood-soaked gauze (also used in several developed countries where the incidence of sPPH is low) can improve the accuracy of blood loss estimation; however, this may require the availability of weight-measuring devices in the labour rooms and wards for prompt measurements.

Expert Recommendations for Treatment and Management of PPH in a Resource-Limited Setting

Although the Malaysia MOH guidelines provide a prophylaxis and treatment plan for PPH (Table 5), considering the differential influence of several patient-intrinsic factors on vaginal delivery versus caesarean section delivery and the availability of these treatments in resource-limited settings, treatment management for PPH following vaginal delivery should be different from that following caesarean delivery (Table 6).

Oxytocin and carbetocin are both recommended as first-line treatments, yet doctors from Malaysia use carbetocin selectively due to the costs associated. However, since carbetocin is heat stable unlike oxytocin, it is still prescribed in hospitals where maintaining a cold chain for oxytocin is a challenge. We suggest carbetocin use for prophylaxis for vaginal deliveries and instrumental deliveries rather than caesarean sections as there are several options available to manage the blood loss in caesarean deliveries than vaginal deliveries.

Amongst the invasive treatments, IUBT can be applied in patients with uterine atony undergoing vaginal delivery to arrest prolonged bleeding. IUBT followed by a B-Lynch suture may be considered in patients with uterine atony and undergoing caesarean delivery (Table 6). If bleeding persists due to retained placenta following vaginal delivery, the clinician should proceed with removal of the placenta. For genital tract trauma cases, suturing should be done at the site of the trauma, and internal iliac artery ligation or hysterectomy should be considered if the bleeding persists after each procedure. For both vaginal and caesarean deliveries, hysterectomy should be considered as the last treatment option if everything else fails.

Recommendations for Managing sPPH using MTP

Massive transfusion protocol (MTP) is a key aspect of sPPH management as it improves the ability to receive blood products promptly and greatly benefits the patient. This is helpful, especially for patients with placenta praevia wherein the blood bank is informed in advance about the possibility of activating the MTP during or after the surgery and constantly thawing the blood products such as the fresh frozen plasma can aid in faster blood transfusion to patients. The MTP should include a ratio of 1:1:1 for packed cells, fresh frozen plasma, and cryoprecipitate, with the first cycle typically consisting of four units each. If haemostasis is not achieved after the first cycle, the second cycle of the MTP can be initiated. For ongoing bleeding, blood investigations such as full blood count and coagulation profile should be monitored every 30 minutes.

Recommendations for Managing PPH in Difficult Pregnancies

Another challenge faced was handling PPH in difficult pregnancies; in such cases, appropriate planning was required to arrange for elective caesarean section deliveries for patients with placenta praevia to minimise blood loss during the procedure. This can be done by implementing adjustments such as making the surgical incision higher up on the abdomen so that the placenta is not affected during the surgery.

Expert Recommendations to Increase Knowledge and Training Among Clinicians to Handle PPH and sPPH

More training should be conducted to increase awareness about identifying the clinical signs and symptoms of PPH among HCPs and encourage them to prophylactic treatment and prompt referral to tertiary hospitals. Junior doctors and hospital staff should be trained to monitor PPH patients closely for clinical signs of hypovolaemia. The importance of emergency hospitalisations due to PPH and prompt attendance to any deterioration in the patient clinical status must be reiterated to all HCPs. Immediate initiation of a red alert once PPH is diagnosed is important for timely management and junior doctors must be encouraged to take the call to flag the situation as and when they deem fit without worrying about the repercussions of being reprimanded if it turns out to be a false alarm.

Despite IUBT being widely available and accessible in Southeast Asia, doctors still lack the skills and experience to carry out the procedures, thereby increasing complications like DIC. However, the lack of trained senior consultants to perform hysterectomies adds to the burden of gynaecological oncology consultants in many hospitals. To combat these shortcomings, resources for education and training should be made available for hospitals in remote areas nationwide to enable these institutions to implement life-saving measures before transferring the patient to a specialist hospital.

Evolving Landscape of Managing PPH and Expert Recommendations for Using Different Haemostatic Agents in PPH Management

Studies in recent years have shown the efficacy of tranexamic acid, fibrinogen concentrate, and rFVIIa in the management of sPPH [61]. These treatments are recommended to be administered in parallel to induce haemostasis [61]. rFVIIa is recommended for controlling bleedings in patients with haemophilia with inhibitors, acquired haemophilia, Glanzmann’s thrombasthenia, congenital factor FVII deficiency and more recently in PPH. rFVIIa has demonstrated effective haemostatic outcomes and low risk of adverse events in real-world studies [62]. Several studies evaluating the efficacy and safety of rFVIIa in sPPH have been conducted in more developed countries, however, there is a lack of studies on the same in the Southeast Asian region [61,63-67].

rFVIIa has been recently approved by the European Medicines Agency (EMA) in 2022 and in Malaysia in 2023 for treating PPH when uterotonics are insufficient to achieve haemostasis. A randomised control prospective study involving women with PPH who were unresponsive to uterotonics showed that the women who received rFVIIa had a decreased need for invasive interventions [68]. rFVIIa was associated with a reduction in the number of patients who needed second-line therapies (about one in three patients; Figure 3), with the occurrence of non-fatal venous thrombotic events in one in 20 patients [68]. rFVIIa use has also been shown to not increase the risk of thromboembolic events in real-world studies [61,63-67]. The use of rFVIIa could be considered prior to definitive surgical treatment, with the aim of minimising blood loss during the surgery. Another ideal use for rFVIIa would be for the reduction of blood loss during the transfer of sPPH patients from smaller hospitals to specialist hospitals for further treatment [22,68].

Strengths and Limitations

A key strength of this scoping review lies in the involvement of local clinicians/experts who provided practice-based insights, ensuring our analysis reflects real-world clinical experiences rather than purely theoretical frameworks. The study's specific focus on resource-limited settings, particularly Malaysia and Southeast Asia, addresses a critical gap in the literature where most PPH research originates from high-resource contexts with limited applicability to developing healthcare systems. Additionally, our review emphasizes the development of practical, implementable recommendations that account for existing infrastructure limitations, workforce constraints, and cultural considerations specific to the regional context. Language limitations may have excluded relevant studies published in local languages, potentially missing important regional insights. The analysis primarily relies on studies from Malaysia and selected Southeast Asian countries, which may limit the generalizability of findings to other resource-limited settings with different healthcare systems, cultural contexts, or economic conditions. The lack of adequate published data makes it difficult to ascertain the challenges faced in the treatment and management of both PPH and sPPH especially in Malaysia.

## Conclusions

This scoping review highlights the significant burden of PPH and sPPH in Southeast Asia, particularly in Malaysia, while revealing a critical gap in reporting and research. The lack of uniformity in treatment management and limited studies on pharmacological interventions in resource-constrained settings underscore the urgent need for standardized protocols. Our expert recommendations propose revised diagnostic criteria for sPPH, emphasizing the importance of clinical signs and symptoms beyond blood loss estimation.

Addressing the burden of PPH and sPPH in Southeast Asia requires a multifaceted approach, including improved reporting, standardized protocols, enhanced training, and the adoption of novel therapies. Future research should focus on evaluating the efficacy and safety of these interventions in the Southeast Asian context to further refine management strategies and improve maternal outcomes.
